# Gestational age assessed by optical skin reflection in low-birth-weight newborns: Applications in classification at birth

**DOI:** 10.3389/fped.2023.1141894

**Published:** 2023-03-28

**Authors:** Gabriela Luiza Nogueira Vitral, Roberta Maia de Castro Romanelli, Zilma Silveira Nogueira Reis, Rodney Nascimento Guimarães, Ivana Dias, Nilza Mussagy, Sergio Taunde, Gabriela Silveira Neves, Carolina Nogueira de São José, Alexandre Negrão Pantaleão, Gisele Lobo Pappa, Juliano de Souza Gaspar, Regina Amélia Pessoa Lopes de Aguiar

**Affiliations:** ^1^ Faculty of Medicine, Universidade Federal de Minas Gerais, Belo Horizonte, Brazil; ^2^ Faculdade Ciências Médicas de Minas Gerais, Belo Horizonte, Brazil; ^3^ Hospital Central de Maputo, Maputo, Mozabique; ^4^ Hospital Sofia Feldman, Belo Horizonte, Brazil; ^5^ Computer Science Department, Universidade Federal de Minas Gerais, Belo Horizonte, Brazil

**Keywords:** infant, low birth weight, premature (babies), small for gestational age (SGA), artificial intelligence, clinical trial, ultrasonography, prenatal

## Abstract

**Introduction:**

A new medical device was previously developed to estimate gestational age (GA) at birth by processing a machine learning algorithm on the light scatter signal acquired on the newborn's skin. The study aims to validate GA calculated by the new device (test), comparing the result with the best available GA in newborns with low birth weight (LBW).

**Methods:**

We conducted a multicenter, non-randomized, and single-blinded clinical trial in three urban referral centers for perinatal care in Brazil and Mozambique. LBW newborns with a GA over 24 weeks and weighing between 500 and 2,500 g were recruited in the first 24 h of life. All pregnancies had a GA calculated by obstetric ultrasound before 24 weeks or by reliable last menstrual period (LMP). The primary endpoint was the agreement between the GA calculated by the new device (test) and the best available clinical GA, with 95% confidence limits. In addition, we assessed the accuracy of using the test in the classification of preterm and SGA. Prematurity was childbirth before 37 gestational weeks. The growth standard curve was Intergrowth-21st, with the 10th percentile being the limit for classifying SGA.

**Results:**

Among 305 evaluated newborns, 234 (76.7%) were premature, and 139 (45.6%) were SGA. The intraclass correlation coefficient between GA by the test and reference GA was 0.829 (95% CI: 0.785–0.863). However, the new device (test) underestimated the reference GA by an average of 2.8 days (95% limits of agreement: −40.6 to 31.2 days). Its use in classifying preterm or term newborns revealed an accuracy of 78.4% (95% CI: 73.3–81.6), with high sensitivity (96.2%; 95% CI: 92.8–98.2). The accuracy of classifying SGA newborns using GA calculated by the test was 62.3% (95% CI: 56.6–67.8).

**Discussion:**

The new device (test) was able to assess GA at birth in LBW newborns, with a high agreement with the best available GA as a reference. The GA estimated by the device (test), when used to classify newborns on the first day of life, was useful in identifying premature infants but not when applied to identify SGA infants, considering current algohrithm. Nonetheless, the new device (test) has the potential to provide important information in places where the GA is unknown or inaccurate.

## 1. Introduction

According to the World Health Organization (WHO), one in 10 newborns is born before 37 weeks of gestation, annually (1, 2). Preterm birth is the leading cause of infant mortality (3, 4). More than 80% of the world's preterm births occur in Asia and Sub-Saharan Africa (5). Low birth weight (LBW) below 2,500 g is also considered a predictor of neonatal mortality and morbidity (6). It is estimated that worldwide, approximately 15% of newborns are born underweight and that more than 90% of these births occur in low- and middle-income countries (7). LBW may be associated with prematurity, intrauterine malnutrition, or a combination of both. Small for gestational age (SGA) newborns are also vulnerable to complications and death and depend on gestational age (GA) for correct classification since they are below the 10th percentile for their age and sex (8).

Accurate information about pregnancy timelines can optimize perinatal and childhood outcomes since this information helps inform immediate decision-making regarding the care of the newborn (9). However, obstetric ultrasounds, widely considered the standard for GA dating, are not always available, especially in low-resource settings (10). In their absence, other antenatal and postnatal methods may be used, such as the date of the last menstrual period (LMP) and assessment of the newborn using the New Ballard score (11). Each of these approaches has its limitations. The LMP reported by the pregnant woman is subject to misremembering and may be unreliable in cases where the woman begins prenatal care late (11, 12). Newborn assessment using physical and neurological maturity scores, such as the New Ballard Score, is commonly used, but with low precision among evaluators, requires trained professionals, and tends to overestimate both the GA and the SGA rate in resource-limited settings (13, 14). Additionally, birth weight, although easily measured, does not distinguish a premature newborn from a SGA newborn. Weights outside the expected range for GA and sex are the best example that, in isolation, this data is not a good estimator of GA (15).

The quality of information about the pregnancy timeline is decisive in the birth scenario. In places where GA is unknown or inaccurate, the risk to the newborn may not be adequately recognized (10). Problems in identifying preterm infants and failures in the classification of SGA are associated with the low availability of obstetric ultrasounds and limited access to essential health technologies (16). It is believed that lives could be saved with adequate care based on the timely identification of premature infants and the appropriate classification of their nutritional status (17).

Addressing these issues is part of public global health policies (18). The development of medical technologies is key to supporting healthcare systems. By mitigating shortcomings in the quality of healthcare services (19), innovative solutions have the potential to save lives (20). In light of this, a new, affordable healthcare device has been developed that estimates GA with an artificial intelligence-based prediction model that uses the photobiological properties of the newborn's skin in combination with clinical variables (21). Assessing 781 newborns any weight, the discrimination between preterm and term newborns *via* the device had a similar area under the receiver operating characteristic curve (0.970, 95% CI: 0.959–0.981) compared with that for LMP- gestational age (0.957, 95% CI: 0.941–0.974) (22). However, the specific evaluation of newborns with LBW is still necessary. This study aims to validate a new test for estimating GA at birth in newborns weighing less than 2,500 g, comparing it with the best available clinical GA. In addition, the aim is to evaluate the test's accuracy in identifying preterm and SGA newborns using the GA calculated by the test and Intergrowth 21st standard.

## 2. Material and methods

### 2.1. Study design and setting

A multicenter, non-randomized clinical trial to verify the accuracy of a new test for predicting GA, with reference standard and blinding. The clinical trial protocol was published on the WHO International Clinical Trials Platform (rBR-3f5bm5) and detailed in a scientific article (23).

The study was conducted in three leading urban centers for perinatal care, two in Brazil and one in Mozambique. The Hospital das Clínicas of the Federal University of Minas Gerais and the Sofia Feldman Hospital are quaternary care centers in Brazil. In Mozambique, the Maputo Central Hospital is considered the largest in the country and is located in the capital. The study was independently approved by the Ethics Committees of the participating institutions, under number 91134218.4.0000.5149 in Brazil and IRB00002657 in Mozambique. Parents signed the informed consent form on behalf of their children.

### 2.2. Participants and eligibility criteria

The criteria for recruitment were live LBW newborns in the first 24 h of life with a GA over 24 weeks and a weight between 500 and 2,500 g. All pregnancies had a GA calculated by obstetric ultrasound up to 24 weeks or by a reliable LMP, as per published clinical protocol (23). The LMP's qualification as reliable was obtained through a direct interview with the woman, with the following criteria: LMP recalled with confidence in the presence of regular menstrual cycles and whose conception occurred at least 2 months after an abortion, childbirth, or the discontinuation of use of hormonal contraceptives (24). Anhydramnios, edema, congenital skin diseases, or chorioamnionitis were the exclusion criteria because of their potential to modify skin structure. The first evaluation took place on February 15, 2019 and the last one on November 12, 2021.

The sample size calculation was informed by the research protocol (23).

### 2.3. Procedures and standards

The examiners were trained according to ISO (International Organization for Standardization) ISO 14155:2011 recommendations for good clinical practices involving human research with medical devices (25). To standardize the procedures for the approach, recruitment, clinical data collection, and examination of the newborn, they remained available in the form of a Standard Operating Procedure (SOP). The error of the optical component was previously measured: intraobserver error 1.97% (95% CI: 1.84–2.11) and interobserver 2.6% (95% CI: 2.1–3.1) (22).

Data collection took place using a paper form and an electronic system. This allowed for a double-check procedure to verify the reliability and validity of clinical data. In addition, the researchers photographed clinical documents that contained information on the LMP and obstetric ultrasound before 24 weeks. The data collected was checked against the information in the photographs. Birth weight was obtained using a digital scale, and the value of the first weighing on the first day of life was considered.

The best clinical GA, used as a reference to assess agreement with the test GA, was obtained by combining the one calculated by reliable LMP with the first obstetric ultrasound before 24 weeks, if any. For this purpose, we used existing information and the Committee on Obstetric Practice American Institute of Ultrasound in Medicine Society for Maternal–Fetal Medicine (9).

### 2.4. The intervention

The test under evaluation in this study is the result provided by an optical device equipped with a processor and a screen for user interaction. In a previous study, the prediction model was developed by adopting ultrasound between 7 and 13 weeks of gestation, with a crow-rump-length measure inclusion criteria (22). The reported GA value is obtained by processing an algorithm that uses the light signal acquired on the newborn's skin and the clinical variables birth weight and the use of antenatal corticosteroids for fetal maturation (ACTMF) (26). Thus, the GA estimated by the prediction model is the test performed in this study, exclusively with LBW newborns (Supplementary File).

**Figure 1:**
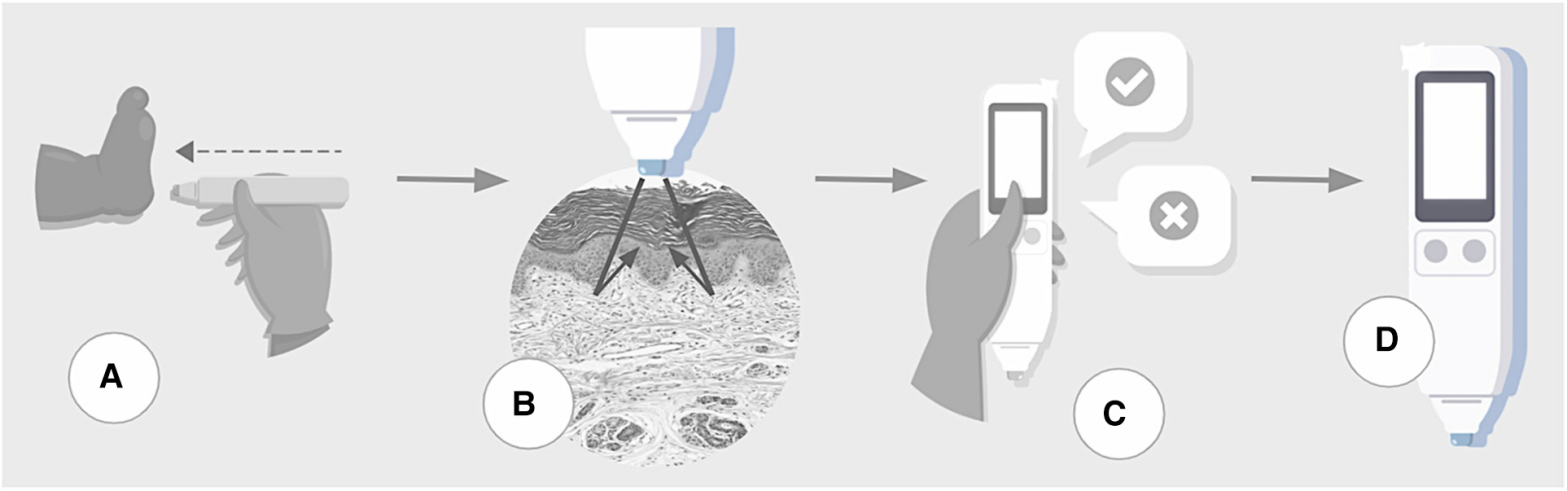
Steps of testing process. (**A**) The device touches the skin over the sole of a newborn. (**B**) The sensor acquires skin maturity by assessing the photobiological properties of the tissue by measuring the reflection portions of the light beam incident on the skin. (**C**) The user inputs clinical data. (**D**) Data processor uses machine learning algorithms to estimate gestational age and classify the infant as preterm or term, small-for-gestational-age or not.

The assessment begins when the examiner touches the sole of the newborn's foot, in the calcaneal region, for a few seconds. The device automatically emits 10 measurements each time it is triggered. The sensor touched the sole three times, following complete disinfection with alcohol (Figure 1). The median of 30 values is the final result of the skin reflection acquisition. In addition, it issues alerts in the event of measurement errors caused by the involuntary movement of the newborn or the examiner. The assessment took place wherever the newborn was, i.e., in an incubator, heated crib, bassinet in the hospital room, or on the mother's lap. Thus, it was possible to perform the test with minimal manipulation and avoid imbalanced clinical conditions.

During the proof of concept step of development (21), the sole was the site of the newborn body with a higher linear coefficient between the skin reflection and GA than others, with the advantage of attending the patient security recommendation for minimum manipulation of newborns. Furthermore, concerning the intervenient effect of humidity, environment temperature and light, and newborn incubator staying, they were removed with enhanced sensor design, achieving a prediction model without environmental variable adjustments (22).

### 2.5. Gestational age prediction model used by the test

The GA prediction model was developed using a machine learning method, eXtreme Gradient Boosting (XGBoost) (27). The problem was addressed as a regression task, where the model predicted the GA of the newborn. In a previous clinical trial, the performance of the generic model reached *R*^2^ 0.878; mean error −1.34 (95% CI: −2.04 to 0.64) days in relation to the reference GA for newborns of any weight (22). The present study is the external validation of the prediction model in scenarios where the available information for calculating GA is not always obstetric ultrasound. As in this study, only LBW newborns were considered, the original model did not generalize well, as in its original training data, most LBW were born preterm. To create the model used in the present study, 326 newborns weighing less than 2,500 g were selected from the original training data. Then, a random data oversampling technique was used to generate a new data sample of newborns with clinical characteristics more similar to those of this new clinical trial. XGBoost was used to generate a new model with this new data and validated in the present study (Supplementary File). In addition to skin reflection, the predictive variables for GA were birth weight and whether ACTMF was used, at any dose or therapeutic regimen. In the case of missing ACTMF information, XGBoost automatically addressed data imputation.

### 2.6. Outcomes

The present study evaluated the immediate outcomes related to the use of GA and its use in the classification of newborns, set forth in the published clinical protocol (23). Postnatal follow-up and outcomes at 72 h were not addressed in this analysis. The primary outcome was the agreement between the GA calculated by the prediction model (test) and the best available clinical GA calculated by obstetric ultrasound before 24 weeks or reliable LMP. The secondary outcomes were the correct identification of preterm newborns before 37 weeks using the GA predicted by the test relative to the best available clinical GA and, moreover, with a margin of error of 1 week. In addition, the correct identification of SGA newborns, according to the GA predicted by the test, gender, and weight below the 10th percentile on the Intergrowth curve 21st (28).

### 2.7. Statistical analysis

Descriptive statistics: the numerical variables were described in terms of mean and standard deviation or median and interquartile range, depending on the nature of their distribution. The categorical variables were described by their absolute and relative frequency. The comparison of subgroups of interest was performed using Pearson's chi-square test for categorical variables and the *T*-test for means or the Mann–Whitney test for quantitative variables.

The intraclass correlation coefficient (ICC) and Pearson's correlation coefficient (R) were used to analyze the agreement between the clinical GA and the GA predicted by the model. Differences between gestational ages were compared using the paired *T*-test of means. Bland-Altman (29) correlation and scatter plots were used. The accuracy of the GA, estimated by the prediction model used to identify preterm and SGA newborns, was evaluated using sensitivity, specificity, positive predictive value, negative predictive value, positive likelihood ratio, negative likelihood ratio, negative likelihood ratio, and ROC curves. The significance level for the hypothesis tests was 5%, and the confidence intervals were calculated as 95%.

Analysis subgroups were created considering the source of information used to calculate the reference GA, denominated the GA-LMP subgroup gathering the newborns with a LMP-based GA and the GA-USB subgroup with newborns with before-to-24 weeks prenatal ultrasound-based GA. The intention was to compare the test results against two different clinical methods currently used to calculate GA at birth. Part of the LMP subgroup was composed of newborns who lacked an obstetric ultrasound examination before 24 weeks, in which case the LMP was classified as reliable. The other part was composed of newborns whose GA reclassification method pointed to the LMP as the reference.

## 3. Results

**Figure 2:**
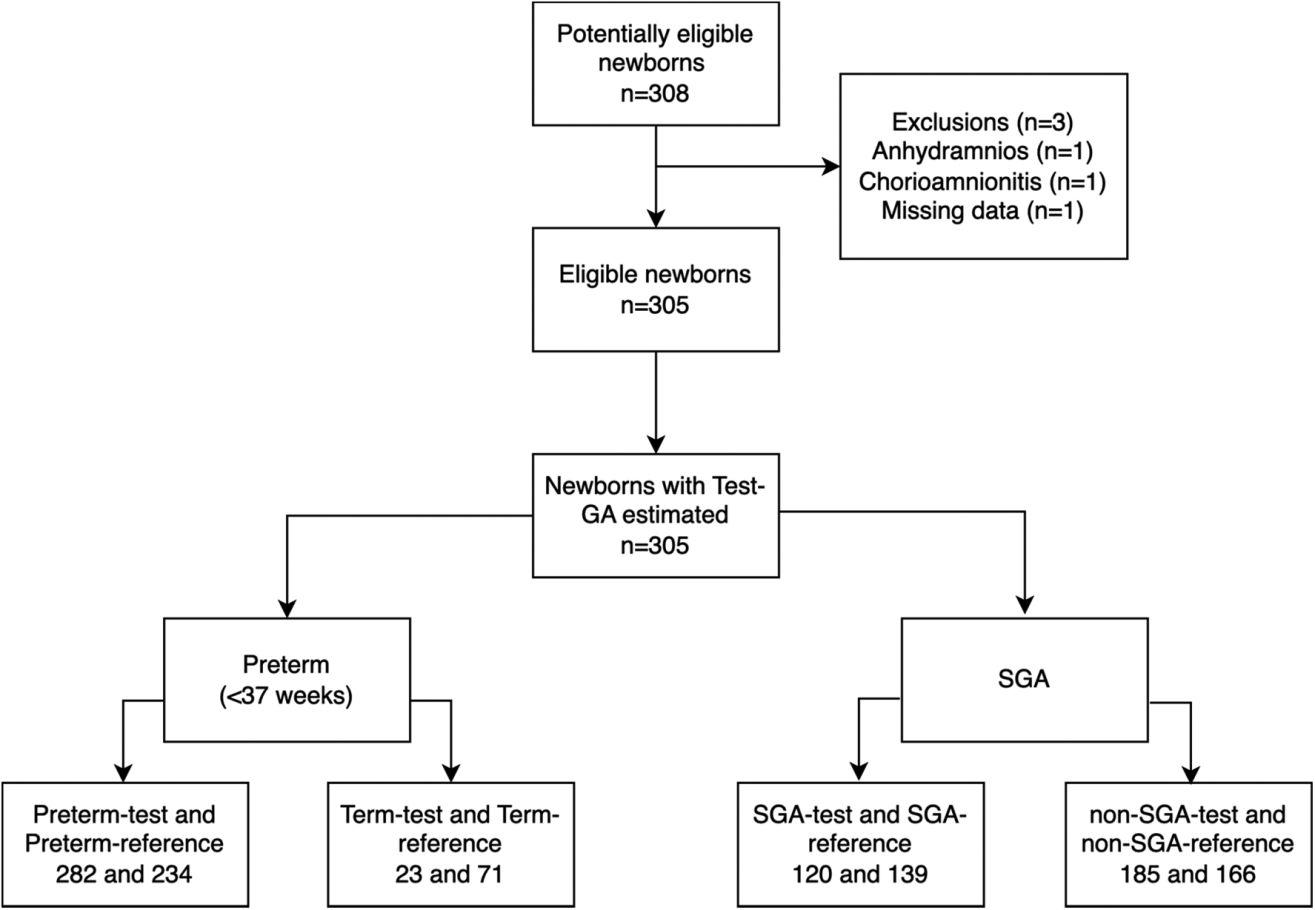
STARD diagram of study participants with the primary outcome results. GA, gestational age; SGA, small for gestational age.

Three of the 308 potentially eligible newborns were excluded, two due to established exclusion criteria and one due to the loss of essential data, Figure 2. The remaining 305 newborns had their skin assessed and were included for analysis.

### 3.1. Description of the women

In total, 260 pregnant women gave birth to 305 newborns. The majority of the pregnant women, 198 (76.2%), had some comorbidity. The most frequently found diseases were hypertensive disorders, corresponding to 136 pregnant women (52.7%), and diabetes, found in 19 pregnant women (7.3%). Among infectious complications, HIV infection occurred in 23 (8.8%) pregnant women and syphilis in 6 (2.3%).

**Figure 3:**
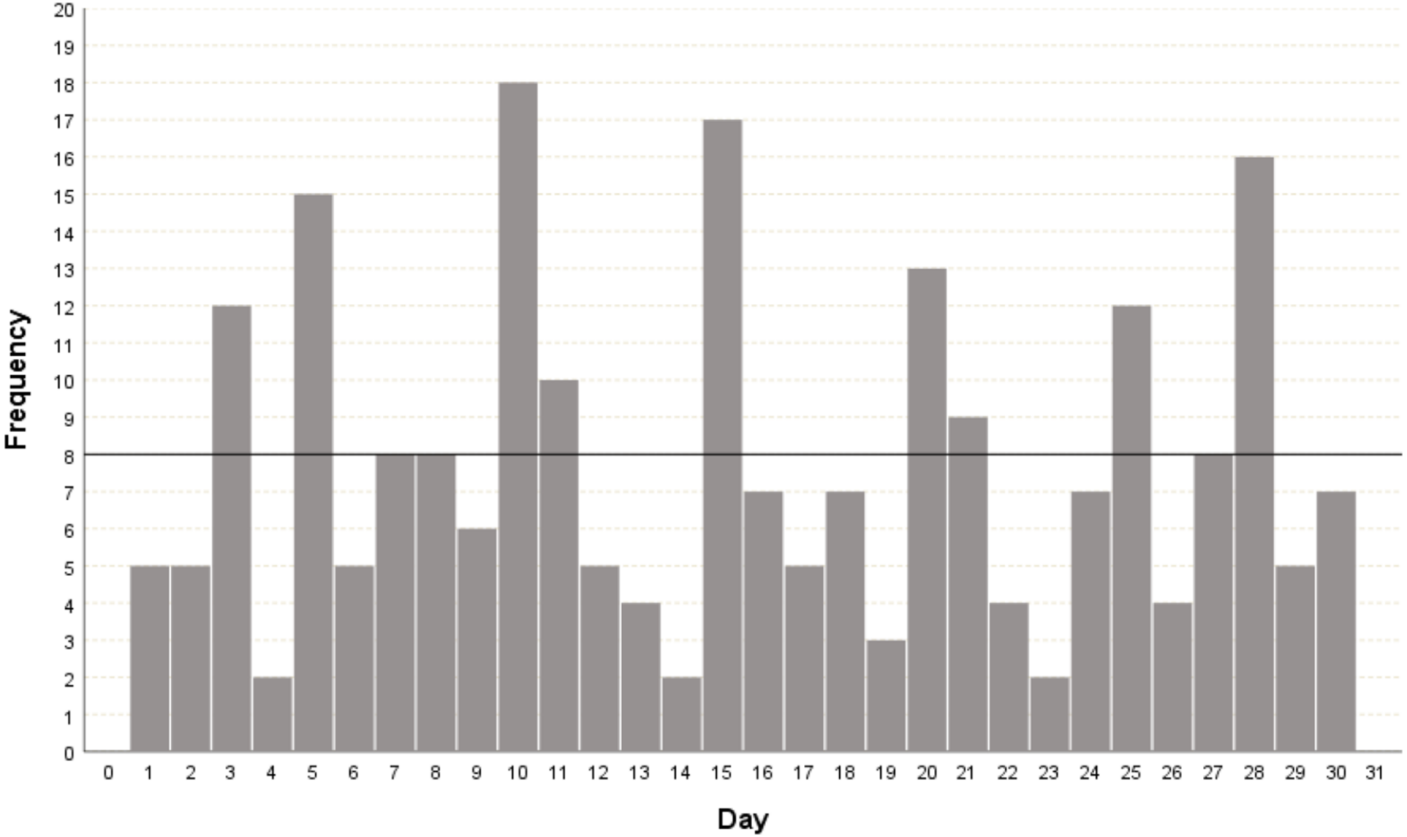
Histogram for the day of the month of the date of the last menstrual period as reported by the pregnant woman. The highlighted black line corresponds to the expected frequency of the day of the LMP reported among the 30 days of the month; that is, it is expected that each day of the month will appear eight times.

The date of the first prenatal visit ranged from 4 to 34 weeks gestation (median 13, IQR 8). In 19 (7.3%) pregnant women, the GA at the beginning of prenatal care was not reported. Regarding the quality of pregnancy dating, among the 235 (90.4%) pregnant women who recalled their LMP, only 168 (71.5%) met the criteria for reliable dating. The evaluation of LMP quality using day preference analysis is shown in Figure 3, revealing the multiples of five-digit preference. Regarding the obstetric ultrasound, 164 (63.1%) pregnant women underwent the examination before 24 weeks (median 13, IQR 6.25) and of these, 100 (61.0%) reported having an ultrasound performed before 14 weeks of gestation.

### 3.2. Description of the newborns

Of the 305 LBW newborns evaluated, 177 (58.0%) were from Mozambique, and 128 (42.0%) were from Brazil. Regarding the dating of GA at birth, in 184 (60.3%), the reference used was the LMP and in 121 (39.7%), it was the obstetric ultrasound before 24 weeks, following consensus choice criteria (2). The clinical characteristics of the newborns are shown in Table 1.

**Table 1:** Clinical characteristics of low-birth-weight newborns included in the study.

Characteristics	Total (*n* = 305)	Subgroup LMP-GA (*n* = 184)	Subgroup USB-GA (*n* = 121)	*p*
Reference gestational age at birth (weeks), average (SD)	34.3 (3.5)	34.5 (3.4)	33.9 (3.7)	0.156*
Preterm, *n* (%)	234 (76.7)	144 (78.3)	90 (74.4)	0.433***
Birth weight (g), median (IQR)	1,930 (687)	1,930 (588)	1,930 (961)	0.564**
Size for gestational age				0.107***
SGA, *n* (%)	139 (45.6)	82 (44.6)	57 (47.1)	
AGA, *n* (%)	155 (50.8)	92 (50.0)	63 (52.1)	
LGA, *n* (%)	11 (3.6)	10 (5.4)	1 (0.8)	
Preterm and SGA, *n* (%)	71 (23.3)	43 (23.4)	28 (23.1)	0.963***
Term and SGA, *n* (*n*%)
ACTFM, *n* (%)	141 (46.4)	71 (38.6)	70 (58.3)	0.001***
1 min Apgar score, median (IQR)	7 (2)	7 (2)	8 (3)	0.003**
5 min Apgar score, median (IQR)	9 (1)	8 (1)	9 (2)	<0.001**
Sex (male), *n* (%)	131 (43.0)	72 (39.1)	59 (48.8)	0.096***
Phototherapy^a^, *n* (%)	26 (8.8)	17 (9.5)	9 (7.7)	0.592***
Incubator accommodation^a^, *n* (%)	218 (71.5)	139 (75.5)	79 (65.3)	0.052***
NICU^a^, *n* (%)	225 (73.8)	141 (76.6)	84 (69.4)	0.161***
Neonatal death until 72 h, *n* (%)	20 (6.6)	14 (7.6)	6 (5.0)	0.360***
Childbirth scenario				<0.001***
Mozambique, *n* (%)	177 (58.0)	149 (81.0)	28 (23.1)	
Brazil *n* (%)	128 (42.0)	34 (19.0)	93 (76.9)	

LMP-GA, gestational age calculated using the last menstrual period; USB-GA, gestational age calculated using prenatal ultrasound before 24 weeks of gestation; GA, gestational age; SGA, small for gestational age; AGA, appropriate for gestational age; LGA, large for gestational age; ACTFM, antenatal corticosteroid therapy for fetal maturation; NICU, neonatal intensive care unit.

* Mean *t*-test.

** Mann-Whitney.

*** Qui-square test.

^a^ At the skin assessment.

The subgroup of newborns whose GA was calculated by the LMP did not differ from the subgroup whose GA was calculated by obstetric ultrasound before 24 weeks in the following aspects: Reference GA at birth, preterm frequency, birth weight, and nutritional status. At the time of testing, the subgroups were also similar regarding the use of phototherapy, incubator accommodation, and admission to a Neonatal Intensive Care Unit (NICU). However, the 1st and 5th minute Apgar scores were lower in the subgroup dated by LMP, as was the use of ACTMF therapy. Regarding the birth scenario, the majority (81.0%) of newborns from Mozambique had the reference GA at birth calculated by the LMP, while the majority (76.9%) of newborns from Brazil had an ultrasound before 24 weeks as a reference.

### 3.3. Primary outcome

The results of the tests for the agreement between the GA calculated by the device (test) and the best available clinical GA calculated by obstetric ultrasound before 24 weeks or reliable LMP are presented in Table 2. In the entire group, the test GA underestimated the reference GA by an average of 2.8 days, with 95% limits of agreement ranging from −40.6 to 31.2 days. In the subgroup of newborns with ultrasound-based GA, the difference was −3.6 days on average, with the 95% limit of agreement (−25.3 to 24.1) days compared to those in the GA-LMP subgroup of −2.2 days, with 95% limits of agreement −46.2 to 37.4. In addition, the GA-USB subgroup of newborns exhibited the highest ICC with the test GA.

**Table 2:** Agreement between test and reference gestational age in low-birth-weight newborns.

	Total (*n* = 305)	Subgroup LMP-GA (*n* = 184)	Subgroup USB-GA (*n* = 121)
Day paired difference with reference GA, average (SD)	−2.8 (16.8) *p* = 0.004*	−2.2 (19.1) *p* = 0.122*	−3.6 (12.4) *p* = 0.002*
ICC with reference GA (95% CI)	0.829 (0.785–0.863)	0.717 (0.622–0.789)	0.928 (0.896–0.950)
Bland-Altman 95% limits for the test GA (days)	−40.6 to 31.2	−46.2 to 37.4	−25.3 to 24.1

LMP-GA, gestational age calculated using the last menstrual period; USB-GA, gestational age calculated using prenatal ultrasound before 24 weeks of gestation; GA, gestational age; ICC, intraclass correlation coefficient.

* Paired *t*-test.

**Figure 4:**
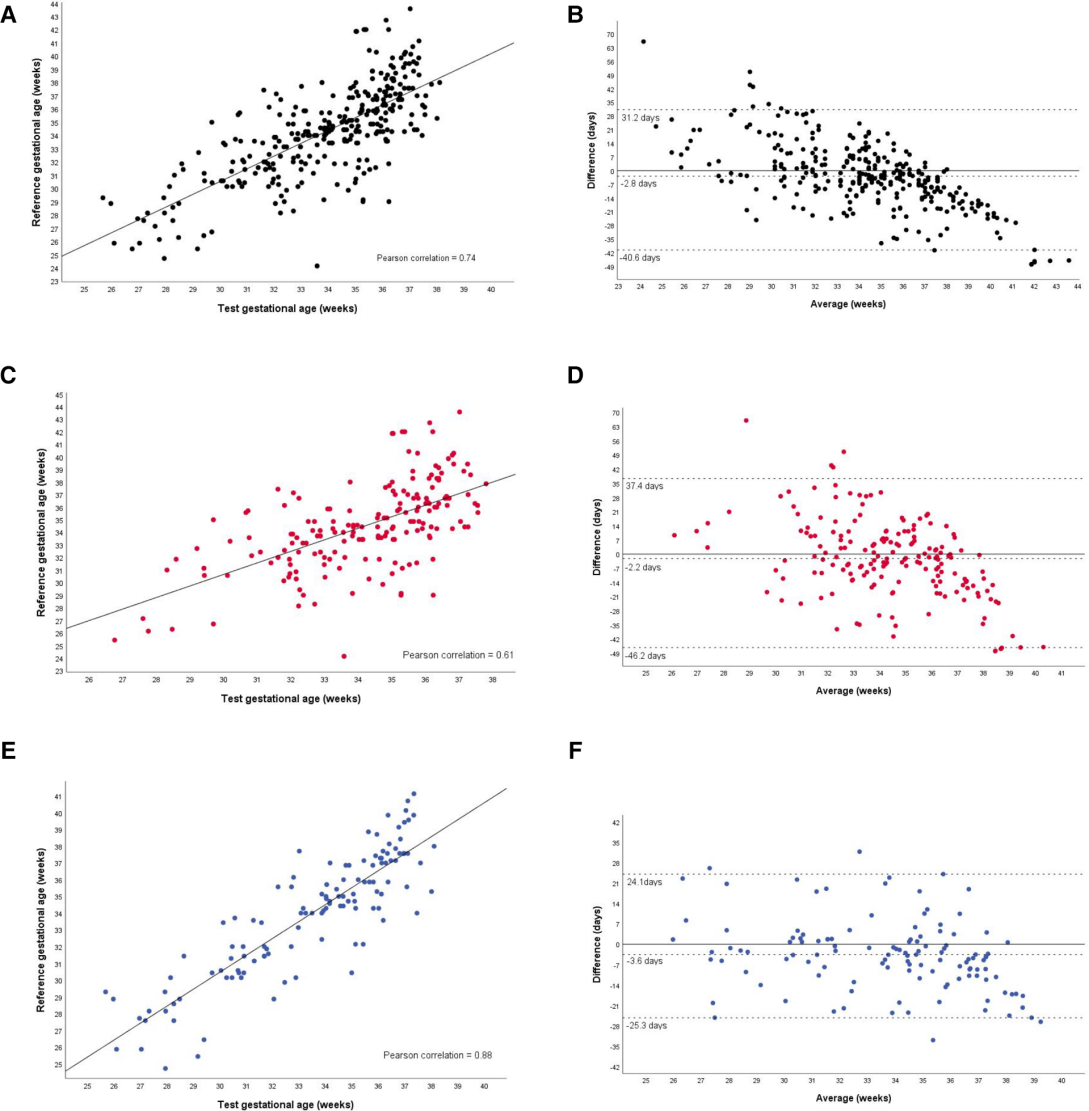
Agreement between the gestational age test and the reference gestational age, in the total group and subgroups. Total group (**A,B**). Gestational age based on the last menstrual period (**C,D**). Gestational age based on prenatal ultrasound before 24 weeks of gestation (**E,F**).

Analyzing the Bland-Altman plots (Figure 4), it can be seen that GA was more frequently underestimated in newborns over 35 weeks of age, both in the total group and in the subgroups.

### 3.4. Secondary outcomes

Considering a 7-day tolerance for the difference between the GA estimated by the test and the reference GA, there was agreement in 129 (42.3%, 95% CI: 37.1%–47.9%) newborns. In the subgroup of newborns with GA calculated by the LMP, the accuracy was 67 (36.4%, 95% CI: 29.3%–43.5%), while in the subgroup dated by the US before 24 weeks, the accuracy was 62 (51.2%, 95% CI: 42.1%–60.3%).

Regarding the discrimination between preterm and SGA infants, the GA predicted by the test discriminated preterm from term with 78.4% accuracy and high sensitivity, detecting 225 out of 234 preterm infants (96.2%), but with low specificity (19.7%) (Table 3). In the GA-LMP and GA-USB subgroups, the test GA showed similar accuracy, considering the overlapping of 95% CI. However, regarding the likelihood ratio, both positive and negative, the test was useful in detecting preterm infants considering the total group and the GA-USB subgroup of newborns.

**Table 3:** Accuracy of the classification of low-birth-weight newborns as premature and small for gestational age.

Classification	Sensitivity % (IC95%)	Specificity % (IC95%)	Accuracy % (IC95%)	VPP % (IC95%)	VPN % (IC95%)	LR+ (IC95%)	LR− (IC95%)
**Total (*n* = 305)**
Preterm	96.2 (92.8–98.2)	19.7 (11.2–30.9)	78.4 (73.3–82.9)	79.8 (77.8–81.6)	60.9 (41.3–77.5)	1.20 (1.06 to −1.35)	0.20 (0.09–0.43)
SGA	51.8 (43.2–60.4)	70.1 (63.6–77.9)	62.3 (56.6–67.8)	60.0 (53.0–66.7)	63.8 (59.1–68.2)	1.79 (1.34–2.39)	0.68 (0.56–0.83)
**LMP-GA subgroup (*n* = 184)**
Preterm	95.8 (91.2–98.5)	15.0 (5.7–29.8)	78.3 (71.6–84.0)	80.2 (78.0–82.3)	50.0 (25.4–74.6)	1.13 (0.99–1.29)	0.28 (0.09–0.81)
SGA	45.1 (34.1–56.5)	66.7 (56.6–75.7)	57.1 (49.6–64.3)	52.1 (43.1–61.0)	60.2 (54.3–65.8)	1.35 (0.94–1.95)	0.82 (0.65–1.05)
**USB-GA subgrup (*n* = 121)**
Preterm	96.7 (90.6–99.3)	25.8 (11.9–44.6)	78.5 (70.1–85.5)	79.1 (75.4–82.4)	72.7 (43.0–90.4)	1.30 (1.05–1.61)	0.13 (0.04–0.46)
SGA	61.4 (47.6–74.0)	78.1 (66.0–87.5)	70.3 (61.3–78.2)	71.4 (60.1–80.6)	69.4 (61.5–76.4)	2.81 (1.69–4.66)	0.49 (0.35–0.70)

SGA, small for gestational age; NPV, negative predictive value; PPV, positive predictive value; LR+, likelihood ratio positive; LR−, likelihood ratio negative; LMP-GA, gestational age calculated using the last menstrual period; USB-GA; gestational age calculated using prenatal ultrasound before 24 weeks of gestation; GA, gestational age.

Regarding the use of the test GA to correctly identify SGA newborns, it did not perform satisfactorily, exhibiting low sensitivity and moderate accuracy in the total group, Table 3. The specificity was 70.1% in identifying non-SGA newborns. Regarding the positive and negative likelihood ratio, the test showed little utility in detecting SGA and non-SGA newborns.

**Figure 5:**
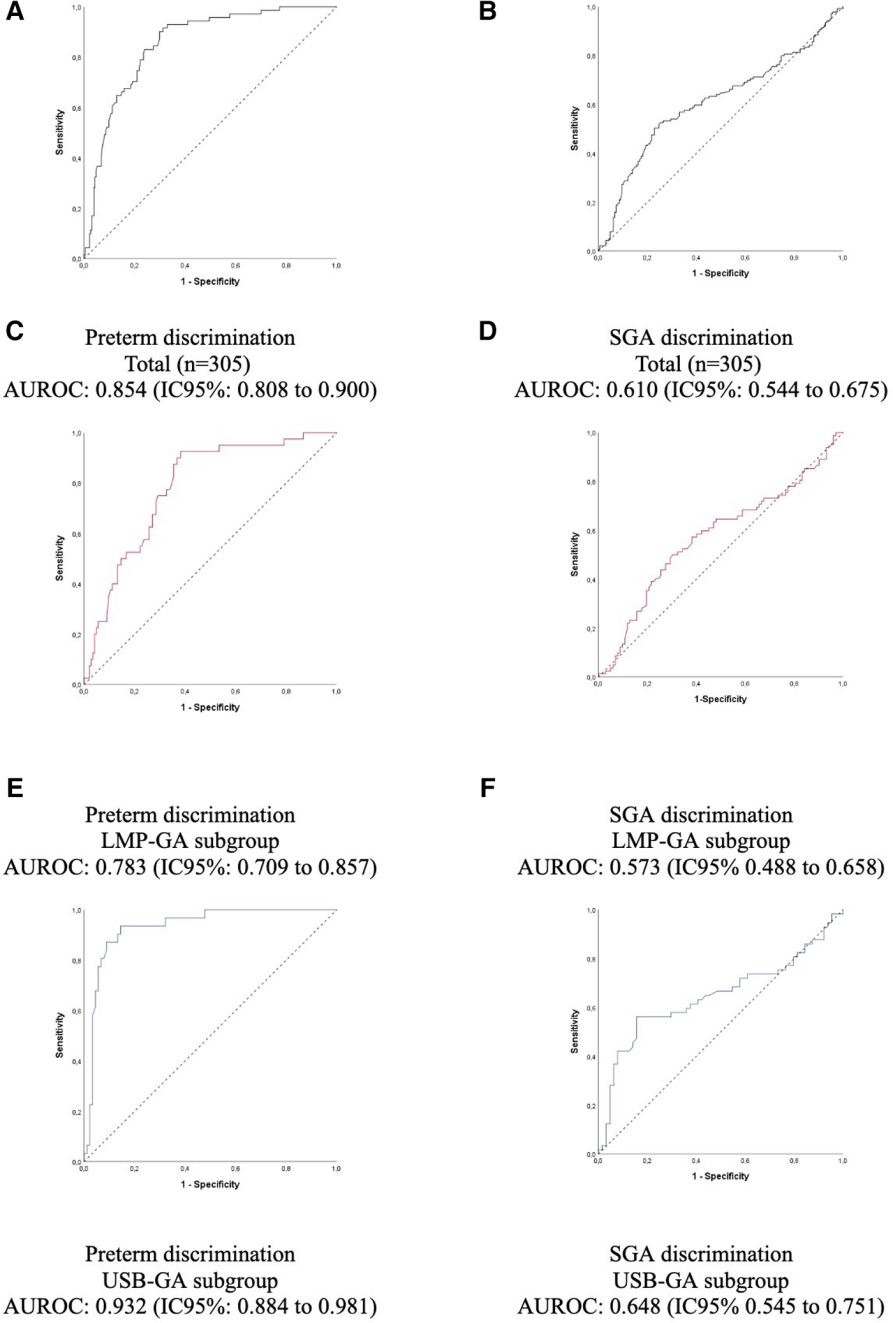
Accuracy of prematurity and small for gestational age with the gestational age predicted by the test (**A,B**), and predicted by the last menstrual period (**C,D**), predicted by the gestational age calculated using prenatal ultrasound before 24 weeks of gestation (**E,F**). AUROC, area under the receiver operating characteristic curve; GA, gestational age; LMP, last menstrual period; SGA, small for gestational age; LMP-GA, gestational age calculated using the last menstrual period; USB-GA, gestational age calculated using prenatal ultrasound before 24 weeks of gestation.

Accuracy analysis *via* ROC curves confirmed the findings. Regarding the detection of prematurity, the area under the ROC curve (AUROC) was high in the total group (0.854), being higher in the GA-USB subgroup compared to the GA-LMP, given that the 95% CIs were not overlapping (Figure 5). For the classification, SGA, not SGA, the area under the curve was low (0.610), with the lower limit of the 95% CI close to the value considered random for the test, 0.544.

**Figure 6:**
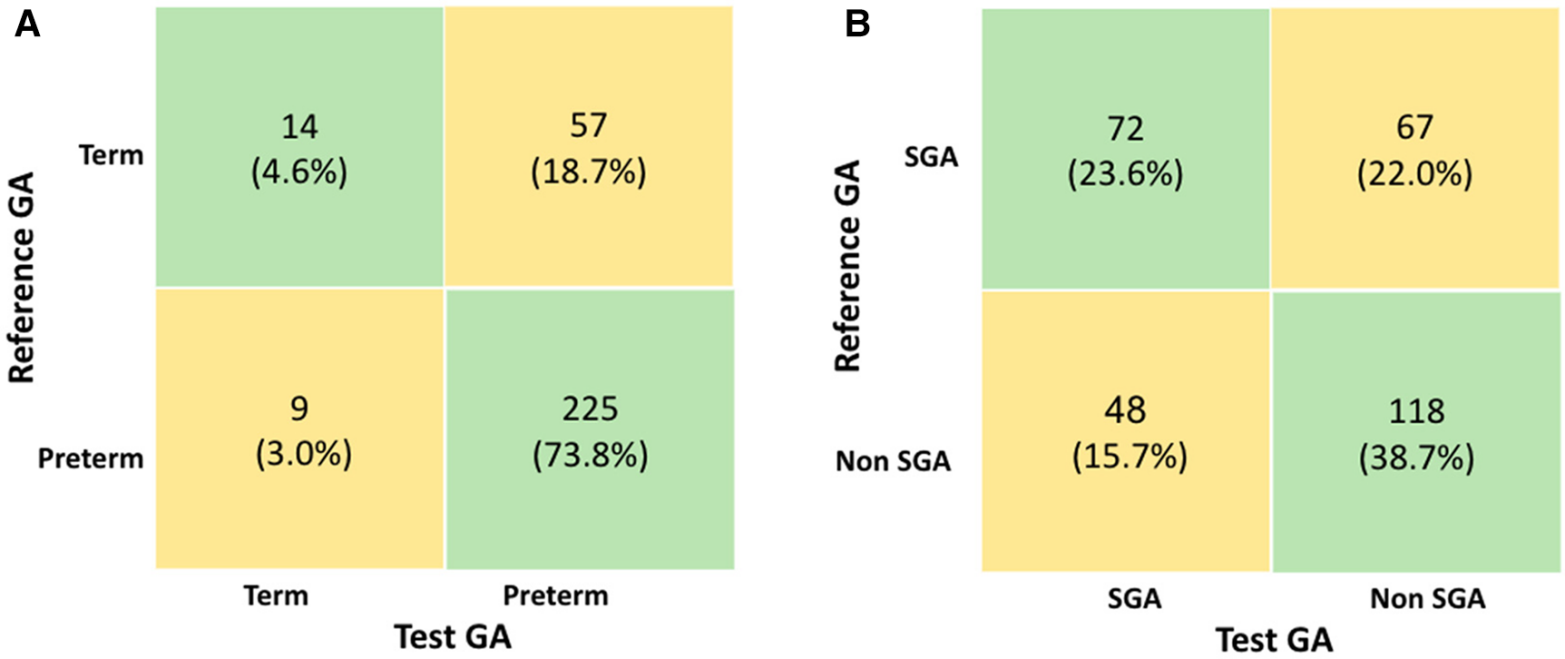
(**A**) Confusion matrix for classification of preterm and (**B**) confusion matrix for classification of small for gestational age newborns. GA, gestational age; LMP, last menstrual period; SGA, small for gestational age.

Confusion matrix: The classification of preterm or full-term newborns using the GA calculated by the test was correct in 239 newborns (78.4%), Figure 6. Fifty-seven (18.7%) full-term newborns by reference GA were reclassified as preterm by the test GA, indicating an overestimation of prematurity. Meanwhile, nine (3%) newborns designated preterm by the reference GA were reclassified as term by the test GA. Among the 66 newborns incorrectly classified by the test, 40 (60.6%) were part of the GA-LMP subgroup, and 26 (39.4%) were part of the GA-USB subgroup.

Regarding the classification of the SGA newborns, the use of the test GA was mainly correct for 118 (38.7%) non-SGA, according to Figure 6. The number of newborns classified as SGA based on reference GA and discordant by the test GA was 67 (22.0%), which means that the test underestimated the true SGA. Among the 115 newborns incorrectly classified by the test, 79 (68.7%) were part of the GA-LMP subgroup, and 36 (31.3%) were part of the GA-USB subgroup.

## 4. Discussion

The main finding of this study was the validation of a test capable of estimating GA at birth in LBW newborns, with a high agreement in relation to the best available reference GA. The crown-rump length measurement *via* ultrasound performed between 7 + 0 and 13 + 6 weeks is considered the most accurate for estimating GA at birth (9). However, to enable analysis in settings with limited access to prenatal care and high-cost technological resources, this study used standardized and acceptable benchmarks (2). Since in previous reports, the GA calculated by LMP has a greater margin of error than that calculated by ultrasound (10), the results were presented in the total group and by subgroups (GA-LMP subgroup and GA-USG subgroup. In the analysis of the subgroup of newborns whose GA was calculated by obstetric ultrasound, the ICC was higher than that of the GA-LMP subgroup. The Bland-Altman limits were also lower. Regarding the agreement between the reference GA and the GA estimated by the test, with a 7-day tolerance, about half of the newborns in the GA-US subgroup were in agreement. These results can be explained because the generic GA prediction model trained in a previous clinical trial used early obstetric ultrasounds as its reference GA (22). In addition, the uncertainties in pregnancy dating by LMP found in this study corroborated previous publications. As described by van Oppernraaij et al. (12), the analysis of memory errors surrounding the LMP in our study revealed a preference for certain days of the month. In the present study, even considering reliable LMP according to references (24), bias may be present. In the analysis, the preference was for multiples of five values. Thus, it is considered that calculating GA is not always trivial and that caregivers must combine good practices with the available data to obtain the best estimate, especially in resource-poor settings (30).

The use of the Bland-Altman limits of agreement and scatterplots to complement the analysis of the test application scenario was also important. In late preterm and full-term newborns, the test underestimated GA more frequently. One possible interpretation is based on the physiological process of skin maturation. In the development phase of the device, the relationships between the maturation of the newborn's skin and the optical properties were studied and it is believed that, as the GA advances, the thickening of the layers of the epidermis and dermis leads to greater light reflection (21, 31, 32). It has been observed that this relationship is directly proportional until approximately 35 weeks of gestation (21), a moment that coincides with the complete development of the epidermis (33). Therefore, it is possible that the test performs better in estimating GA in preterm newborns than in term newborns.

Although the test tended to underestimate GA, it was able to identify premature newborns at the 37-week cutoff with good accuracy. The high AUROC was primarily due to the test GA's high sensitivity in classifying premature infants. Consequently, the advantage is that most premature newborns will be identified (sensitivity), avoiding their false classification as term newborns when in fact they are preterm. However, the low specificity may require other methods of confirming prematurity, since part of the full-term newborns will be classified as premature (false-positive).

The determination of GA by maturity scores after birth is well documented in the literature (11). The New Ballard score is currently used but was not evaluated in the present study. An analysis of the accuracy of this score in SGA newborns showed an overestimation of the GA of 0.7 (95% CI: 1.1–0.2) weeks in relation to the reference (14). However, such a study differs from ours in that it excluded newborns whose GAs were calculated by LMP and early obstetric ultrasound with differences greater than 2 weeks. It is also worth mentioning that a recent study comparing postnatal methods such as foot length, anterior lens vascularization, and bedside assessment by neonatologists concluded that none of the methods were individually useful for estimating GA when compared to early obstetric ultrasound (34).

The test GA assessed along with birth weight, gender, and the INTERGROWTH-21st growth curve (35) was not useful for differentiating between SGA and non-SGA since about one-third were incorrectly classified. Fetal growth is dynamic and depends on maternal and fetal factors as well as the proper functioning of the placenta. It is believed that maternal diseases frequently found in this sample may also affect this process since they affect fetal growth (36, 37). In this study, the recruited newborns displayed a high frequency of SGA, prematurity, and neonatal death within 72 h, with a quarter of them being preterm and SGA simultaneously. In addition, pregnant women in the present study had a high prevalence of chronic diseases, such as hypertension and diabetes, as well as infectious diseases, such as HIV and syphilis, in relation to the prevalence expected among pregnant women in general (38). Thus, identifying newborns with growth below the 10th percentile expected for their GA in a high-risk group exceeded the potential of the test GA, as it is based on skin maturity and not an assessment of nutritional status. Additionally, the choice of the INTERGROWTH-21st standard may have affected the results since it may be associated with an overestimation of SGA (39).

Regarding the benefits and limitations of the new test that estimates GA, since this study is a clinical trial, its usefulness was verified in settings that closely resemble those in which the equipment may be advantageous. Despite its potential to provide important information in places where GA is unknown or inaccurate, the test underestimated by −2.8 days on average compared to reference GA by established methods such as LMP and obstetric ultrasound (9). This may result in unnecessary interventions in some newborns because it overestimates prematurity, which was also exhibited by the high sensitivity values with low specificity. Even if it places an initial burden on the provision of care, the use of the test indicates timely care for truly premature infants who might otherwise be neglected in the absence of any gestational chronology.

Furthermore, the GA among the subgroups of newborns calculated by the LMP was statistically similar to the GA estimated by the test. This seems to be favorable for using the new test in scenarios where there is no information available for calculating GA. Under such conditions, the test can provide information equivalent to that of a reliable GA-LMP. It is hoped that such information will contribute to securing appropriate, prompt care and the transfer of the newborn, when necessary, to specialized centers. Another advantage is that the device was developed in such a way that it is not affected by chromophores in the newborn's skin, such as melanin, previously reported by our group (31).

Machine learning is being widely adopted as a new approach to health data analysis (40–42). Such approaches have been used in other studies to estimate GA or predict preterm birth. The AMANHI Group used machine learning models to estimate postnatal GA using the physical anthropometric variables associated with maturity, such as skin texture, of 7,428 newborns. The best model estimated the GA with 15.7 days of error in relation to the reference GA, calculated by obstetric ultrasound before 20 weeks. It should be noted that all of the systematically-tested machine learning models underestimated GA by 4–5 days in SGA newborns, while they overestimated GA by 1 day in AGA children. In the same study, the sensitivity of the New Ballard score was very low for detecting premature infants (9%) (42), pointing to a challenge yet to be overcome in this clinically highly vulnerable group. Rittenhouse et al. used a set of maternal and neonatal variables accessible at the time of delivery to predict GA at birth compared with the ultrasound before 14 weeks of gestation (41). For this, the study considered maternal information associated with SGA, such as maternal hypertension, twin pregnancy, and HIV seropositivity. The best machine learning model used to estimate GA and predict prematurity excluded the New Ballard score and maintained the LMP along with the set of variables, correctly classifying 94% of newborns (41). This approach may be used in future analyses to improve the use of the test GA to classify SGA by reusing data from the present study. Fung et al. developed machine learning algorithms to estimate fetal GA from ultrasound measurements taken during the second and third trimesters of pregnancy, improving the accuracy of antenatal dating (40).

The new test analyzed here proposes not to use any obstetric ultrasound parameters, but rather data that are easy to obtain in low-tech settings. Even so, the test, which is based on processing information from the reflection of light off of the newborn's skin in addition to clinical variables such as weight and ACTMF, was unable to discriminate between the small and healthy from those who are pathologically small. Although this study did not address fetal growth restriction, most SGA newborns are pathologically small (36). It is possible that future approaches involving maternal conditions associated with restricted fetal growth could compose algorithms for predicting SGA, similar to the study by Rittenhouse et al. (41). However, the test showed promise for screening in settings without reliable antenatal information for classifying preterm newborns, who need increased care. Improving access to obstetric ultrasounds, considered the gold standard for gestational age, remains a priority. Obstetric ultrasounds are scarce in low-income countries.

The potential bias of this study is mainly associated with sub-optimal pregnancy dating since the inclusion criteria admitted obstetric ultrasound examinations before 24 weeks and even the absence of any ultrasound as long as the LMP was reliable. However, this made it possible to conduct the study in settings where the test would be most useful. A previous study evaluated the performance of the algorithm by comparing the predicted value with the best GA, adjusted by ultrasound before 14 weeks (22). Another potential bias was to validate the prediction model that was originally described for newborns of any weight in LBW newborns, with mathematical adaptations. In addition, ACTMF information may not be easily accessible at the time of testing, particularly in low-resource settings. However, this information was absent for only one newborn in the present study. Even so, all recruited participants were included in the analysis because the algorithm is able to handle the absence of that data. Additionally, this clinical trial was not randomized, since studies involving the validation of new diagnostic equipment require comparative methods in the same individual to whom the test is applied (42).

Newborns weighing less than 2,500 g are a group more vulnerable to perinatal complications, including death, and were therefore chosen as potential beneficiaries of the new test. Postnatal assessment methods are helpful in identifying at-risk newborns and preventing mortality and morbidity outcomes (34). In addition to this comparison, the maturity tests are ideally performed between 24 and 72 h of life, which delays the GA assessment. Moreover, such scores suffer neurological abnormalities in newborns without vitality that need resuscitation and may be influenced by growth restriction or nutritional alteration (14). Therefore, the new medical device (test) may be helpful at birth, as soon as possible, after resuscitation steps once the newborn is stable, irrespective of neurological scores. However, future analyses comparing the new test with existing postnatal methods may point out their advantages and disadvantages. Until then, it is hoped that a healthcare professional using the new device will be able to detect premature infants by simply capturing the skin's reflection and entering the clinical information necessary for the algorithm to make a prediction. Regarding the potential to support public policies, adjusting preterm birth rates is an asset for using this technology in LMIC. Improve counting of preterm births is a priority in planning and monitoring actions to achieve national Sustainable Development Goals (2). In a recent meta-analysis study, our group reported higher values in the proportion of preterm birth, ranging from 1% to 3% when the LMP is the reference compared to obstetric ultrasound until 24 weeks of gestation (43). This way, accessible and more accurate approaches are welcome to obtain a reliable GA at birth.

In conclusion, the new test was able to estimate GA at birth in LBW newborns, demonstrating high agreement with the best available reference GA. The GA estimated by the equipment under test, when used to classify newborns on the first day of life, was useful in identifying premature infants but not in identifying SGA infants. Nonetheless, the equipment has the potential to provide important information in places where the GA is unknown or inaccurate, especially in low-income countries.

## Data Availability

Data are available upon reasonable request and after anonymization to allow the sharing of data ethically and legally, thus preserving the confidentiality of the persons who participated in this study.
