# Supplementary material for: Gestational age assessed by optical skin reflection in low-birth-weight newborns: Applications in classification at birth
Source: Front Pediatr. 2023 Mar 28;11:1141894. doi: 10.3389/fped.2023.1141894 (PMC10086374; doi:10.3389/fped.2023.1141894)
Supplement: Supplementary file 1 [file Table1.docx]

***Supplementary Material***

Gestational Age Assessed by Optical Skin Reflection in Low-Birth-Weight Newborns: Applications in Classification at Birth

**Gabriela Luiza Nogueira Vitral, Roberta Maia de Castro Romanelli, Zilma Silveira Nogueira Reis*, Rodney Nascimento Guimarães, Ivana Dias, Nilza Mussagy, Sergio Taunde, Gabriela Silveira Neves, Carolina Nogueira de São José, Alexandre Negrão Pantaleão, Gisele Lobo Papa, Juliano de Souza Gaspar, Regina Amélia Pessoa Lopes de Aguiar**

*** Correspondence:** zilma.medicina@gmail.com

# Supplementary Data

When using machine learning algorithms to generate predictive models, data is divided into two subsets: the training and test sets. The training set is used to build the model, while the test set is used to validate the efficacy of the model.

In a previous study (1), the XGBoost algorithm was used to generate a model to predict gestational age, and then the prediction was used to classify newborns in pre-term and term. The model learns from the input data listed in the row named "Original" in Table A. Considering both term and preterm neonates, 42% were small-for-gestational-age.

**Table A:** Data distribution for different datasets in terms of weight and gestational age.

|  | **Term** | | | **Preterm** | | |  |
| --- | --- | --- | --- | --- | --- | --- | --- |
|  | **Weight** | | **Subtotal** | **Weight** | | **Subtotal** | **Total** |
| **Dataset** | **<2500g** | **>=2500g** |  | **<2500g** | **>=2500g** |  |  |
| Original | 18 | 397 | 415 (53%) | 308 | 58 | 366 (47%) | 781 |
| Validation | 71 | 0 | 71  (23%) | 234 | 0 | 234  (77%) | 305 |
| Original filtered by weight | 18 | 0 | 18  (5.5%) | 308 | 0 | 308  (94.5) | 326 |
| Oversampled | 144 | 0 | 144  (32%) | 308 | 0 | 308  (68%) | 452 |

In this study, the data distribution differs substantially from the aforementioned, as presented in line "validation" of Table A. In this dataset, all babies weighed less than 2500g, and 77% were preterm.

Models trained in very different distributions from the validation data may have problems with generalization. There are different ways of dealing with this problem, including resampling techniques (2). These techniques change the input data distribution to make it more faithful to the real-world scenario distribution and include undersampling and oversampling.

There are two popular techniques to perform oversampling: randomly or by generating synthetic instances based on the values of the dataset (3). In the medical context, clinicians opt for random sampling, as synthetic instances could not make much sense. As its name implies, random oversampling selects a set of instances randomly and replicates them in the training set, in a way that the data distribution is more faithful to the target distribution.

In this paper, we chose to build a new model designed exclusively for small babies, although the distribution of the original dataset could have also been changed. For that, we started by selecting the 326 small-for-gestational-age neonates from the original training set (line "Original filtered by weight" in Table A). We then applied oversampling, generating the dataset described in line "Oversampled" of the column "Dataset" of Table A.

After preliminary experiments and a study from the literature, the proportion of small babies born a term that was small for gestational age was set as 30% of, and those born preterm 70%. This was the data used to generate the model used to predict the gestational age of the newborns in the validation dataset.

**References**

1. Reis Z, Guimarães R, RMdC R, JdS G, Neves G, PdJH N, et al. Newborn skin maturity model for gestational age prediction: a clinical trial for a novel medical device validation. 2022.

2. Kinney MV, Lawn JE, Howson CP, Belizan J. 15 Million preterm births annually: what has changed this year? : BioMed Central; 2012. p. 1-4.

3. Harsurinder Kaur, Husanbir Singh Pannu, and Avleen Kaur Malhi. 2020. A Systematic Review on Imbalanced Data Challenges in Machine Learning: Applications and Solutions. ACM Comput. Surv. 52, 4, Article 79 (July 2020), 36 pages. https://doi.org/10.1145/3343440.
